# Diet Supplementation, Probiotics, and Nutraceuticals in SARS-CoV-2 Infection: A Scoping Review

**DOI:** 10.3390/nu12061718

**Published:** 2020-06-08

**Authors:** Fabio Infusino, Massimiliano Marazzato, Massimo Mancone, Francesco Fedele, Claudio Maria Mastroianni, Paolo Severino, Giancarlo Ceccarelli, Letizia Santinelli, Elena Cavarretta, Antonino G. M. Marullo, Fabio Miraldi, Roberto Carnevale, Cristina Nocella, Giuseppe Biondi-Zoccai, Cristiano Pagnini, Sonia Schiavon, Francesco Pugliese, Giacomo Frati, Gabriella d’Ettorre

**Affiliations:** 1 Department of Clinical Internal, Anesthesiologic and Cardiovascular Sciences, Sapienza University of Rome, 00185 Rome, Italy; fabio.infusino@uniroma1.it (F.I.); massimo.mancone@uniroma1.it (M.M.); francesco.fedele@uniroma1.it (F.F.); paolo.severino@uniroma1.it (P.S.); fabio.miraldi@uniroma1.it (F.M.); cristina.nocella@uniroma1.it (C.N.); 2 Department of Public Health and Infectious Diseases, Sapienza, University of Rome, 00185 Rome, Italy; massimiliano.marazzato@uniroma1.it (M.M.); claudio.mastroianni@uniroma1.it (C.M.M.); giancarlo.ceccarelli@uniroma1.it (G.C.); letizia.santinelli@uniroma1.it (L.S.); 3 Department of Medical-Surgical Sciences and Biotechnologies, Sapienza University of Rome, 04100 Latina, Italy; elena.cavarretta@gmail.com (E.C.); antoninogm.marullo@uniroma1.it (A.G.M.M.); roberto.carnevale@uniroma1.it (R.C.); giuseppe.biondizoccai@uniroma1.it (G.B.-Z.); sonia.schiavon@uniroma1.it (S.S.); fraticello@inwind.it (G.F.); 4 Mediterranea Cardiocentro, 80133 Naples, Italy; 5 Department of Gastroenterology and Digestive Endoscopy, Azienda Ospedaliera San Giovanni Addolorata, 00184 Rome, Italy; cpagnini@hsangiovanni.roma.it; 6 Department of General Surgery and Surgical Specialities “Paride Stefanini”, Sapienza, University of Rome, 00185 Rome, Italy; francesco.pugliese@uniroma1.it; 7 IRCCS NeuroMed, 86077 Pozzilli (IS), Italy

**Keywords:** supplementation, probiotics, nutraceuticals, SARS-CoV-2, COVID-19

## Abstract

The severe acute respiratory syndrome coronavirus 2 (Sars-CoV-2) global pandemic is a devastating event that is causing thousands of victims every day around the world. One of the main reasons of the great impact of coronavirus disease 2019 (COVID-19) on society is its unexpected spread, which has not allowed an adequate preparation. The scientific community is fighting against time for the production of a vaccine, but it is difficult to place a safe and effective product on the market as fast as the virus is spreading. Similarly, for drugs that can directly interfere with viral pathways, their production times are long, despite the great efforts made. For these reasons, we analyzed the possible role of non-pharmacological substances such as supplements, probiotics, and nutraceuticals in reducing the risk of Sars-CoV-2 infection or mitigating the symptoms of COVID-19. These substances could have numerous advantages in the current circumstances, are generally easily available, and have negligible side effects if administered at the already used and tested dosages. Large scientific evidence supports the benefits that some bacterial and molecular products may exert on the immune response to respiratory viruses. These could also have a regulatory role in systemic inflammation or endothelial damage, which are two crucial aspects of COVID-19. However, there are no specific data available, and rigorous clinical trials should be conducted to confirm the putative benefits of diet supplementation, probiotics, and nutraceuticals in the current pandemic.

## 1. Introduction

Since its first detection in Wuhan, China, December 2019, the severe acute respiratory syndrome coronavirus 2 (SARS-CoV-2) has had a dramatic worldwide diffusion [1]. Accordingly, the World Health Organization (WHO) declared a pandemic on 11 March 2020. The clinical severity spectrum of SARS-CoV-2 may range from asymptomatic cases to severe pneumonia resulting in acute respiratory distress syndrome and sometimes leading to multi organ failure (MOF). More recently, new insights concerning the disease course are emerging. Some patients are initially characterized by dyspnea and hypoxemia, which can rapidly progress to a mild respiratory syndrome needing O_2_ therapy. In contrast, other patients rapidly progress to acute respiratory distress syndrome (ARDS), sometimes evolving in septic shock, metabolic acidosis, coagulation dysfunction with disseminated intravascular coagulation (DIC), and multiple organ dysfunction syndrome (MODS). Thus, COVID-19 has emerged as a multifaceted, multi-system, multi-organ disorder which produces its pathogenic effects through a quite ubiquitous target at the level of multiple organs. Notably, all age groups are susceptible to the virus, and elderly patients with comorbidities are more likely to experience a severe illness. As of 29 May 2020, over 5,800,000 confirmed cases have caused more than 360,000 deaths [2].

Scientific research efforts are focused on producing a vaccine quickly, because it would be the best way to halt the pandemic. However, despite several groups working on it, it is difficult to guarantee the marketing of a safe product in a short time. Other efforts are actually trying to understand the pathogenesis of COVID-19, which would be extremely useful to identify a specific and more efficient therapy. Although there is no clear clinical evidence for effective antiviral drugs, several antivirals targeting the molecular pathways of SAR-CoV-2 have been used worldwide [3], but only remdesivir has shown to be effective in shortening the time to recovery of hospitalized COVID-19 patients [4]. Also, a short-term use of corticosteroids to inhibit the cytokine cascade and to prevent disease progression toward a severe form could be considered for patients with severe COVID-19 pneumonia [5]. The widely used treatment with chloroquine and hydroxychloroquine (often in association with macrolides) has shown mixed benefits in the available studies and may even be harmful according to some authors, due to cardiac toxicity [6,7]. Taking into account these results, the WHO firstly suspended the studies launched on chloroquine and hydroxychloroquine [8]. Nevertheless, there would seem to be a need for general clarification. Indeed, since several concerns were raised with respect to data and analyses performed at the moment we are proofreading our manuscript the article was retracted and the WHO resumed the studies concerning chloroquine and hydroxychloroquine. In reason of a disproportionate and aberrant immune response able to drive COVID-19 to the related ARDS and, in some patients, to fibrosis and widespread lung damage, tocilizumab, a humanized anti-interleukin-6-receptor (IL-6R) monoclonal antibody that inhibits interleukin-6 (IL-6) signaling, is currently under investigation in several clinical trials [9]. 

Preliminary data have created interest in anti-cytokine therapy to counteract the inappropriate immune responses as a beneficial therapeutic strategy, and accordingly, the Italian Drug Agency (AIFA, Agenzia Italiana del Farmaco) has also recently approved a clinical trial (Sobi.IMMUNO-101) in which a combination of emapalumab (a monoclonal antibody toward IFN-γ) and anakinra (a recombinant human interleukin-1 receptor antagonist) will be synergistically administrated to COVID-19 patients with the hope to induce a rapid serologic and subsequent clinical improvement.

Besides a pharmacological therapy, many people during this period are wondering if some non-pharmacological substances used to “strengthen their defenses” against common winter infections can be useful. 

The pharmacological properties of natural compounds have gained increasing attention in the field of alternative and coadjutant therapeutic approaches to several diseases. Accordingly, the food industry is focused on bioactive substances contained in foods or natural products termed nutraceuticals, which can bring health benefits, besides their intrinsic nutritional values, especially in the treatment of chronic diseases. Moreover, these compounds are characterized by negligible side effects in comparison with traditional pharmacological therapies, so that consumers lean towards their use for health promotion. Regarding the usefulness of non-pharmacological substances, no specific clinical studies for Sars-CoV-2 infection are available yet. However, we will review the possible role of probiotics, nutraceuticals, and diet supplementation in SARS-CoV-2 viral infection. 

## 2. Methods and Design

This scoping review was designed in keeping with the best reviewing practices. Specifically, we searched PubMed for articles on diet supplementation, probiotics, or nutraceuticals for the prevention or treatment of SARS-CoV-2 infection or COVID-19. Specifically, the following string was last run on 16 April 2020: (sars-cov-2 OR covid-19 OR “coronavirus-associated disease 2019”) AND (probiotic* OR nutraceutic* OR diet* OR supplement*). Studies were selected if reporting on original in vitro, in vivo, or human studies, and key study subjects, interventions, and outcomes were systematically collected. All reviewing tasks were performed by two independent reviewers (M. Mancone and P. Severino), with divergences solved after consensus.

## 3. Probiotics

Since the first US case of COVID-19 showing atypical symptoms, diarrhea and other gastrointestinal (GI) manifestations have attracted more attention in the scientific community [10]. 

SARS-CoV-2 infects human cells through the binding of its spike proteins (S) to Angiotensin-Converting-Enzyme-2 (ACE2) [11]. ACE2 is highly expressed in AT2 lung cells but also in esophagus epithelial cells and enterocytes in the ileum and colon [12]. TMPRSS2, a protein responsible for the priming of the viral S protein (necessary for entry into the host cell) is also highly expressed in absorbent enterocytes [13].

SARS-CoV-2 RNA has been detected in the stool of COVID-19 patients [10,14]. A histological study by Fei Xiao et al. performed on the intestinal mucosa of a patient with stool positivity reported infiltrating plasma cells and lymphocytes with interstitial edema in the epithelium of stomach, duodenum, and rectum. Furthermore, the presence of the viral host receptor ACE2 was demonstrated in the cytoplasm of gastrointestinal epithelial cells, while the viral nucleocapsid protein was visualized in the cytoplasm of rectum, duodenal, and gastric epithelial cells [15].

In this last study, GI colonization appeared to be tardive with respect to respiratory infection, and in more than 20% of infected patients, viral RNA was present in stool even after negativization in the respiratory tract. However, this partially contrasts with the report of Wang et al. that showed how the less common GI symptoms like diarrhea, nausea, vomiting, and abdominal discomfort had an early and mild onset compared to the respiratory symptoms [16].

Similar evidence from studies on the 2003 first SARS coronavirus epidemic indicated gastrointestinal tropism, since the virus was found in gastrointestinal biopsies and stool, even in healed patients, which may partially justify the intestinal symptoms, reappearance, and transmission of the disease [17].

An experimental study on mutant mice with ACE2 deficiency demonstrated the role of this receptor in the regulation of innate immunity, preservation of the gut microbiome, regulation of the intestinal amino acid balance, and production of antimicrobial peptides. These functions were independent from the renin angiotensin system, and transplantation of the altered microbiota from ACE2 mutant mice into germ-free wild-type hosts could increase the propensity for serious colitis [18].

Therefore, most of the data suggest that SARS-Cov-2 is more likely transmitted via the respiratory route, but many findings suggest that the intestine could have a relevant role both in the disease pathogenetic evolution and as a possible route of infection. 

Based on the intestinal findings, it is possible to assume that viral replication in the intestine determines an exponential increase in the viral load in the digestive mucosa. This mechanism could lead to a loss of barrier integrity with an imbalance of the microbial flora and its metabolites, determining important consequences on the immune system, which could lead to a strong production of cytokines. 

**Figure 1:**
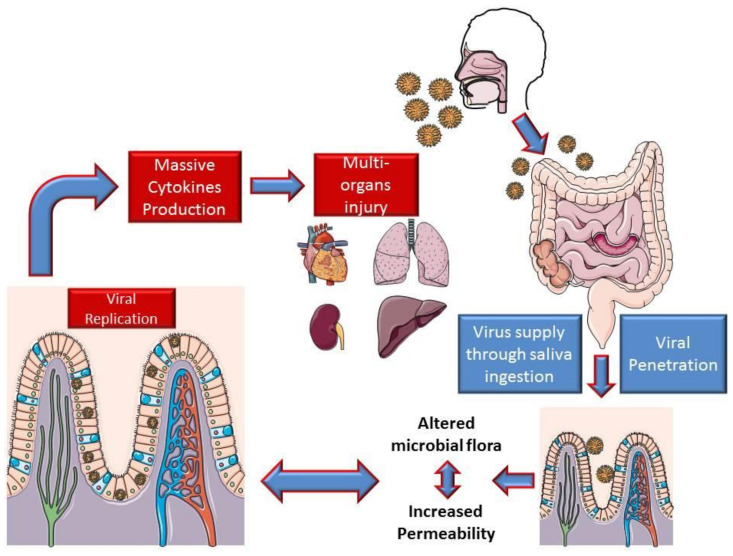
Hypothesis on the mechanism of intestinal involvement in coronavirus disease 2019 (COVID-19).

This may in part justify the appearance of ARDS and MOF following interstitial pneumonia (Figure 1).

Classically, viral infections have been considered a bidirectional process involving, exclusively, the host cell and the virus, with no participating external factors other than the host immune system. In the last decade, thanks to the wide knowledge provided by metagenomic analysis, viral infections, like many other medical occurrences, have been associated with the so-called “microbiota revolution”, which is the tendency to link many pathologic conditions to the intestinal microbiota and its alteration. The term “microbiota” refers to the complex community of microorganisms that stably colonize the mucosal surfaces of the human body. Such microbes constitute a key factor in health and disease, because of their essential metabolic and immunomodulatory functions, as well as for their protection against pathogens [19,20,21]. Particularly, commensal bacteria have resulted to play an essential role in shaping the host immune systems as well as in triggering its responses in both health and disease conditions [22,23]. The presence of an intimate relationship between the host immune system and the microbiota has been primarily evidenced in the gastrointestinal tract by the determination, in germ-free mice, of many immunological defects such as small Peyer’s patches and mesenteric lymph nodes, reduced amount of T helper 17 (Th17) cells, and deficiency in regulatory T cells [24]. These defects reverse, within weeks, after the acquisition of intestinal bacteria from normally colonized mice [25]. Further evidence has shown that the gastrointestinal tract microbiota is able to modulate neutrophils migration and function [26] as well as to influence the differentiation of T cells into Th1, Th2, Th17 helper cells, or T regulatory (Treg) cells [27], which may be linked in turn to tolerance or immune reactions against different luminal bacteria. Compared to the study of the intestinal microbiota, that of the lung microbiota is still at the beginning, but some observations support the role played by commensal bacteria in the lung in immune tolerance maintained by subpopulations of alveolar macrophages and dendritic cells [28]. Such cell types, by inducing the generation of Tregs [29] and releasing prostaglandin E2 (PGE2), tumor growth factor-beta (TGF-β), and interleukin-10 (IL-10) [28], exert immunomodulatory functions.

In murine models, the presence of specific bacterial taxa as well as a general increase of bacterial biomass in the lungs has been associated with the development of Treg cells two weeks after birth [30], while the introduction of commensal bacteria in germ-free mice has been shown to reduce the strong immune response induced by the intranasal administration of ovalbumin (OVA) [31]. In human lungs, bacteria belonging to the phylum Bacteroidetes have been associated with the decrease of lung inflammation [32], while the bacterial genera Prevotella and Veillonella have been implicated in Th17 cell-mediated immune responses [33].

A synergistic interplay between the microbiota and the human host occurs when the microbial communities are characterized by a balanced state, known as eubiosis. Perturbations of such a condition, called dysbiosis, could lead to the loss and/or the dysregulation of the normal functions provided by the microbiota and constitute a pivotal driver for both infectious and non-infectious diseases [34]. To date, a wide range of local and systemic diseases, comprising inflammatory bowel disease, obesity, allergic disorders, atopic dermatitis, autism, colorectal cancer, and diseases affecting both the higher and the lower respiratory tracts, have been associated with microbial dysbiosis [35,36,37,38,39]. In particular, in recent years, the interaction between the intestine and the respiratory system, both for homeostasis maintenance and in disease pathways, has been individuated, and the term “gut–lung axis” was coined to refer to this mutual interaction [40].

Although dysbiosis is often characterized by a multifactorial etiology, one of its main causes is represented by infections carried out by invading pathogens, most commonly by viruses. Studies investigating the interaction between viruses and the microbiota have shown that commensal bacteria, through different mechanisms, are able to regulate or are regulated by invading viruses, thereby leading to harmful or beneficial effects on the host [41,42,43,44,45]. Consensus has been reached that viral infections, including those sustained by influenza viruses, alter the commensal microbiota in both the gastrointestinal and the airway tracts of the host, causing alterations of the microbiota–host relationship, which is a key element in determining infection-related disease. Different studies have evidenced that, in the upper respiratory tract, the influenza virus infection is associated with decreased colonization by health-promoting bacteria as well as with enrichment in potentially harmful microbes. For example, it has been reported that the nasopharyngeal microbiota of patients with viral respiratory tract infections is significantly enriched in bacterial pathogens such as *Haemophilus influenzae*, *Staphylococcus aureus*, *Streptococcus pneumoniae*, and *Moraxella catarrhalis*, [46] while the colonization of the health-associated genus *Prevotella* results to be decreased [46,47]. Although contradictory findings are reported in the current literature for the gut microbiota, resulting from differences in experimental conditions, concerning virus subtypes and doses, experimental animal models, age, diet, and lifestyle of the investigated subjects, a general decrease in the richness of bacteria belonging to the phylum Firmicutes, mainly *Lactobacilli*, has been associated with viral infections and, in particular, with influenza viruses [48,49,50]. One of the postulated mechanisms leading to influenza-associated commensal microbiota dysregulation involves the altered delivery of IFN-γ (a type II IFN) by a subset of lung-derived T cells expressing CC chemokine receptor 9 in the intestine and the subsequent stimulation of epithelial cells to produce IL-15, which induces a Th17-mediated immune response [51]. Although no direct evidence is currently available about the association between SARS-CoV-2 infections and microbial dysbiosis in both the gut and the respiratory tract, the presence of symptoms like diarrhea, nausea, vomiting, and abdominal discomfort, as well as the determined tropism of SARS-CoV-2 for enterocytes [14,15,52], suggests that interactions between this new β-coronavirus and the gut microbiota are possible.

To date, while effective therapies or vaccines to prevent and fight respiratory virus infections are available for influenza and adenoviruses, no effective therapies are available for other respiratory viruses such as those responsible for common cold and the new β-coronavirus SARS-CoV-2. The need to rapidly contrast respiratory viral infections together with the large amount of time and money necessary for the development of vaccines challenge the development of alternative and safe therapies able to reduce the risk of such infections. In this context, the use of probiotics could represent a promising tool in the field of clinical research. Probiotics consist of alive organisms that, when administered in sufficient amounts, confer positive benefits to patients, and their potential clinical utilization has been proposed in numerous pathologic conditions [53]. Although solid evidence is still lacking, many experimental and clinical studies support the possible role of different probiotic microorganisms in protecting the host against viral infections, comprising those responsible for colds and flu [54,55,56,57,58,59].

A recent Cochrane meta-analysis, including 12 randomized controlled trials (RCT) with a total of 3720 subjects, demonstrated that probiotics were able to reduce the number of acute upper respiratory tract infections, the mean duration of disease, antibiotic administration, and cold-related school absences compared to a placebo, although the quality of evidence was low [60].

The exact mechanism(s) of the antiviral activity of probiotics is not completely clear and it likely involves multiple concomitant steps. In particular, the potential therapeutic effect of probiotic bacteria against viral infections could be exerted at three different levels implicating a direct interaction with the virus: (1) by reinforcing the mucosal innate immune response; (2) by reducing intestinal permeability; and (3) by affecting the systemic acquired immune response through a regulatory and anti-inflammatory effect.

Virus attachment to a host cell represent an essential step in viral infection, so probiotic bacteria may inhibit it by directly binding the virus, thus inhibiting the infectious process. It has been reported that lactobacilli are able to bind and inactivate viruses through adsorptive and/or trapping mechanisms. Bacterial strains of *Lactobacillus paracasei*, *Lactobacillus rhamnosus*, as well as *Lactobacillus plantarum* can interact with the envelop of vesicular stomatitis virus (VSV), directly trapping the virus [61]. An inhibiting mechanism involving a direct interaction between bacterial cell wall components and herpes simplex virus type 2 has been also suggested for *Lactobacillus brevis* CD2 strain [62]. Furthermore, exopolysaccharides from *Lactobacillus* species were demonstrated to completely suppress the production of adenovirus-5 in vitro [63].

Lactobacilli, as well as other probiotics, have been reported to possess an immunomodulatory ability and protect from virus infections by enhancing cytokine antiviral responses in respiratory and immune cells and in the intestinal mucosa [63,64,65,66,67]. Oral administration of *L. brevis* in mice protected the animals from influenza infection through the enhancement of antiviral IFN-α as well as an augmented production of specific-IgA antibodies against the virus [68]. *L. plantarum* significantly reduced the titers of human H1N1 and avian influenza H7N9 viruses in mouse lungs after a lethal viral challenge and increased the mean number of days and rates of survival of the infected mice [69].

Interestingly, intranasal administration of lactobacilli showed to be protective against virus respiratory infections, encouraging innate immune responses directly in the airway epithelium [70]. In addition, *L. paracasei*-fed mice showed a lower incidence of influenza A H3N2 infection, associated with a reduced infiltration of inflammatory cells in the lungs and a faster virus elimination [71]. Bacteria belonging to the *Bifidobacteria* group exert protective effects against influenza virus infection. After lethal influenza A (H1N1) infection, a strong stimulation of humoral and cellular immunity, associated with lower levels of proinflammatory IL-6 production and an increase in survival rate of mice receiving *Bifidobacterium bifidum,* with respect to the control group, was observed [72]. Another important preventive action of probiotics against the progression of viral infections could be mediated by the enhancement of the mucosal intestinal barrier that in turn may prevent virus spreading in the sub-mucosal compartment and in the systemic circulation. A multi-strain probiotic mixture has demonstrated a preventive effect on intestinal inflammation onset in a mouse model of spontaneous ileitis, mediated by the stimulation of TNF release from epithelial cells and decreased permeability [73,74]. *L. rhamnosus* GG has shown a beneficial effect in the treatment of infections by enhancing intestinal permeability, with the stimulation of mucin expression and the regulation of proliferation/apoptosis of epithelial cells, both in experimental models and in clinical studies [75,76].

An interesting antiviral activity exerted by probiotics is related to their ability to modulate the immune system towards anti-inflammatory pathways. *Lactobacillus gasseri* exhibits antiviral activity against respiratory syncytial virus (RSV); in fact, oral administration of this probiotic in mice causes a reduction of the RSV titer in the lungs. Moreover, the expression of pro-inflammatory mediators in the lungs due to RSV infection decreased, while interferon-stimulated genes were upregulated by *L. gasseri* treatment [77]. A reduction of virus-induced inflammation was also exerted by a strain of *L. plantarum* in mice after acute infection by pneumonia virus (PMV), a rodent pathogen that induces inflammation and is related to the respiratory syncytial virus [78]. *Bifidobacterium animalis* subspecies *lactis* Bl-04, in an experimental rhinovirus infection, showed to reduce the levels of the pro-inflammatory cytokine IL-6, as well as to reduce the nasal lavage virus titer [79]. Similarly, the administration of this probiotic in the respiratory tract of mice infected with PVM, increased mice survival and reduced the levels of IL-6, whose suppression was demonstrated to be a critical feature of the protective mechanism. It was recently reported that COVID-19, similarly to SARS-CoV, is characterized by a dramatic inflammatory response induced by a cytokine storm associated with increased disease severity [80,81]. Patients needing intensive care present higher plasma levels of many cytokines such as IL-6, IL-1, IP-10, MCP-1, MIP-1A, and TNFα [82] with respect to non- intensive care unit (ICU) subjects, suggesting the likely involvement of a highly pro-inflammatory condition in the disease progression and severity. Furthermore, a large infiltration of inflammatory cells has been observed in the lungs of severe COVID-19 patients [83,84]. These aberrant pathogenic cells, together with inflammatory monocytes, may reach the lungs, causing an immune injury with consequent respiratory disability and increased mortality. The modulation of the cytokine cascade exerted by probiotics may represent a therapeutic approach for severe infections, making it licit to hypothesize that probiotics administration could influence the immune response in patients affected by COVID-19, thus preventing or mitigating the exacerbated inflammatory processes that lead to death.

Although solid evidence for probiotics utilization in the treatment of COVID-19 is still lacking, their complementary use may be proposed, as already stated by China’s National Health Commission and National Administration of Traditional Chinese Medicine in the “Diagnostic and therapeutic guidance for 2019 novel coronavirus disease (version5)” [85]. In particular, probiotic supplementation was suggested as a complementary treatment of gastrointestinal symptoms such as diarrhea and to reduce the risk of secondary infections due to microbial translocation in severe COVID-19 cases [86]. Despite the fact that direct evidence of the possible effect of probiotics on SARS-CoV-2 infection is not available [87], a number of suggestions indicate that this resource could represent a complementary tool to decrease SARS-CoV-2-related inflammation and favor the recovery of intestinal mucosa damage by modulating the gut microbiota. In fact, recent studies suggest that SARS-CoV-2 induces an acute intestinal inflammatory response via ACE2 and transmembrane serine protease 2, characterized by mucosal infiltration of macrophages, neutrophils, and T-cells. [88]. For these reasons, probiotic use could have a beneficial role in patients with COVID-related gastro-intestinal symptoms and in those with mild–moderate systemic symptoms without respiratory impairment and help prevent disease progression. As speculated, it is possible that probiotics may play a role in preventing the cytokine storm and related ARDS or MOF in high-risk individuals with established SARS-CoV-2 infection, but the opposite may also be true, and the lack of sufficient available evidence should be considered. Similarly, in patients with severe disease and in critically ill patients, probiotic administration needs to be cautiously evaluated, and important safety concerns need to be considered when administering bacterial supplements [89]. In fact, it is not known whether the administration of micro-organisms to patients with critical disease conditions could lead to any type of injury, such as an exacerbation of inflammation. A final possible setting is disease prevention, especially for high-risk patients (immunosuppressed and elderly patients with comorbidities), “strategic” people (i.e., health care professionals, workers with extensive public contact), or subjects with suspected COVID-19 waiting for a clear diagnosis and/or who came in contact with a COVID-19-positive subject. The rationale for the possible prophylactic use of probiotics in SARS-CoV2 infection is linked to its ability to preserve a healthy status in the gut-associated lymphoid tissue (GALT) as well as eubiosis, which is necessary to actively fight the entry of the virus into gut cells [90]. The enhancement of the innate defenses may be probably useful in COVID-19 prevention or in the very early phase of the infection. However, it is not known whether the administration of various types of microorganisms to healthy individuals can increase the risk of contracting SARS-CoV-2 infection. Large clinical trials are needed to elucidate these hot topics and identify the most useful and safe setting for probiotics administration. Actually, three registered trials to evaluate probiotics administration to COVID-19 patients are ongoing (Table 1).

## 4. Nutraceuticals

Chronic respiratory diseases are associated with the development of systemic inflammation and oxygen stress resulting in endothelial dysfunction together with increased platelet aggregation and enhanced coagulation. All these factors are involved in the pathophysiology of respiratory diseases, leading to irreversible endothelial dysfunction [94].

It has been previously demonstrated that patients with community-acquired pneumonia (CAP), the most common infection-related cause of death in developed countries, display increased platelet activation mediated by the activation of NOX2, the main enzymatic source of cellular reactive oxygen species (ROS) production, with consequent decrease of flow-mediated dilation (FMD) and NO bioavailability [94,95,96]. Moreover, these patients disclose an ongoing pro-thrombotic state, as suggested by increased plasma levels of F1+2, a marker of thrombin generation, and lower levels of protein C (PC) and activated PC (aPC) [94,95].

In severe forms of COVID-19, it has been proposed that a severe widespread alveolar and interstitial inflammation extends to the pulmonary vasculature. Intra-pulmonary inflammation might negatively modulate a severe local vascular dysfunction including micro-thrombosis and hemorrhage, resulting in pulmonary intravascular coagulopathy (PIC).

Accordingly, the reduced endothelial function, that is an early subclinical stage of vascular alteration, could favor the development of a severe form of this pathology, contributing to increased pulmonary, cardiovascular, and renal complications.

The immune system is the second system most affected by COVID-19 after the respiratory system. An increase in systemic interleukins, chemokines, and tumor necrosis factor-α (TNF-α) has been observed during the rapid progression phase of COVID-19 [80]. These changes correspond to the characteristics of a cytokine release syndrome (CRS) in which IL-6 contributes to many of the key symptoms. The role of IL-6 in COVID-19 patients has been highlighted in a recent retrospective multicenter study [97] showing that circulating IL-6 levels were higher in COVID-19 deceased patients compared to discharged subjects. These results suggest that the “cytokine storm syndrome”, activated by the virus could be a clinical predictor of fatal outcome in these patients. The activation of endothelial cells and the ensuing vascular dysfunction are other typical features of severe CRS. Indeed, typical markers of endothelial activation are often elevated in the serum of patients with CRS. This indicates that the endothelium plays an important role in the pathophysiology of CRS, both by amplifying the inflammatory response and by contributing to clotting and eventually to a thrombotic disease, both in the venous and in the arterial circulations [98].

In addition to the inflammatory process, the mechanisms accounting for clotting and vascular changes may also include oxidative stress. In particular, NOX2-derived ROS are implicated in clotting and platelet activation, promoting thrombin generation and platelet aggregation or impairing artery dilatation.

Thus, oxidative stress and inflammation are closely interrelated and form a vicious feed-forward cycle during atherogenetic plaque progress. Indeed, according to the oxidative stress theory of atherosclerosis, the progression of the atherosclerotic plaque, with plaque rupture, clotting, and ensuing atherothrombosis, is dependent upon artery inflammation [99].

**Figure 2:**
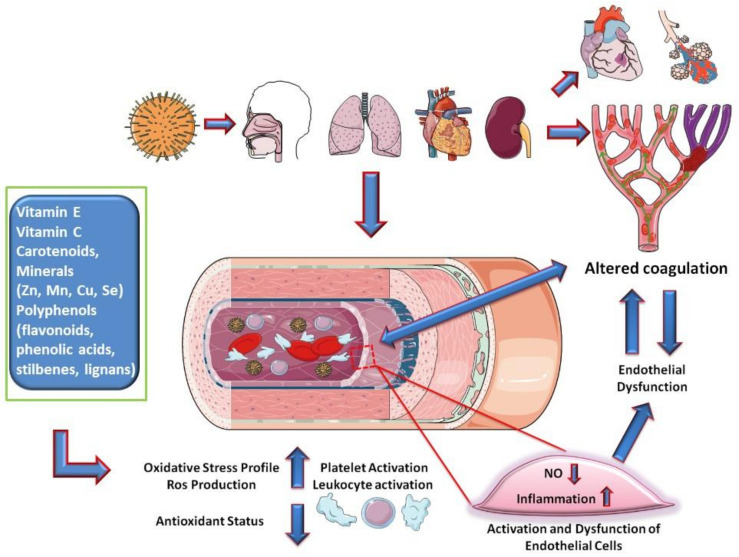
Hypothesis on the mechanisms of endothelial involvement in COVID-19.

Based on these considerations, nutraceuticals, defined as substances that may include isolated nutrients, dietary supplements, diets, and herbal products, could play a role in preventing the phenomena of the inflammatory cascade and hypercoagulation by exerting their anti-inflammatory and antioxidant activities. Among nutraceuticals, vitamin E, vitamin C, carotenoids, and some minerals (Zn, Mn, Cu, Se) and polyphenols (flavonoids, phenolic acids, stilbenes, lignans) provide medical or health benefits by a synergistic effect, maintaining a proper redox homeostasis (Figure 2)

In particular, a diet rich in polyphenols is able to reduce and prevent cardiovascular disease. [100]. Moreover, the protective effect of polyphenols includes the reduction of oxidative stress resulting from the downregulation of NADPH oxidase or from an antiplatelet and anticoagulant function, as indicated by the reduction of platelet aggregation and the suppression of the activity of thrombin and the production of factor Xa [101,102]. Moreover, flavonoids could increase endogenous platelet-derived nitric oxide and decrease superoxide production [103] and might enhance the endothelial synthesis of NO, induce NO-dependent relaxation in isolated arteries, and activate NO signaling pathways in endothelial cells, thus improving the endothelial function. Finally, some polyphenols exert anti-inflammatory activities by modulating cytokine production and by promoting the expression of pro-inflammatory genes [104], and an antiviral effect that has been already reported against several viruses [105].

Given this premise, it is reasonable to consider oxidative stress- and inflammation-mediated endothelial dysfunction as a therapeutic target for Covid-19. Among polyphenols, curcumin could be a potential treatment option for patients with Covid-19. Utomo et al. conducted a study using molecular docking with target receptors including SARS-CoV-2 protease, the receptor binding domain (RBD) of spike glycoprotein, and the protease domain (PD) of ACE2 which are believed to participate in virus infection [106]. They demonstrated that curcumin could bind to the target receptors of SARS-CoV-2, supporting the use of this molecule for preventive or prophylaxis treatments of virus infections, including SARS-CoV-2. Moreover, a combination of three (phyto-) nutrients such as vitamin C, curcumin, and glycyrrhizic acid promotes interferons production and regulates the inflammatory response, suggesting that the combination of these molecules may be helpful in regulating the immune response to combat SARS-CoV-2 infections [107].

Finally, Runfeng et al. tested in vitro the antiviral activity of Lianhuaqingwen (LH), a Chinese patent nutraceutical composed of 13 herbs [108], and they found that LH is able to inhibit SARS-CoV-2 virus replication and markedly reduce the mRNA levels of pro-inflammatory cytokines including TNF-α, IL-6, CCL-2/MCP-1, and CXCL-10/IP-10).

In conclusion, since accumulating evidence suggests that nutraceuticals exert beneficial effects against vascular diseases counteracting oxidative stress and inflammation, it is reasonable to speculate their possible use in the setting of COVID-19.

Regarding the illustrated inflammatory hypothesis, new recent considerations on TNF blockers deserve a mention. Further promising and potentially effective therapies for COVID-19 are the anti-TNF antibodies infliximab and adalimumab, which have also shown a good safety profile [109]. The rationale of their use is related to the demonstrated presence of TNF in the serum and in some tissues of COVID-19 patients, where it promotes a phlogistic response [110]. For several inflammatory diseases, anti-TNF therapies are approved by the FDA and commonly used (e.g., rheumatoid arthritis or psoriasis). Studies on rheumatoid arthritis have shown that blocking TNF stops the cytokine cascade, decreasing the levels of adhesion molecules and vascular endothelial growth factor (VEGF) involved in the pathways of augmented vascular permeability [111,112]. Also, some preclinical studies on RSV and influenza virus in mice suggest a positive response to anti-TNF therapies [113]. Some authors suggested that infliximab or adalimumab administration could be preferentially destinated to COVID-19 patients with moderate disease but at high risk to develop a severe and advanced illness [109,114]. Actually, a single registered RCT evaluating adalimumab for COVID-19 treatment is in progress (ChiCTR2000030089). Main nutraceuticals and supplements with potential role in countering COVID-19 are listed in Table 2.

## 5. Supplementation

### 5.1. Vitamin C

Vitamin C is a water-soluble vitamin able to provide electrons, acting as antioxidant and as a cofactor for regulatory enzymes. In particular, it facilitates the production of cortisol, catecholamines, and vasopressin. Vitamin C is fundamental for both the innate and the adaptive immune system. Vitamin C has a role in the epithelial as well as endothelial barrier function, maintains vasodilation, and reduces proinflammatory modulators [115]. Vitamin C has crucial roles in the improvement of phagocytosis, chemotaxis, and production of ROS, decreasing necrosis and tissue damage [116].

The role of ascorbic acid in modulating the immune system has been studied extensively since the second half of the last century. As early as 1978, a study by J.G. Atherton et al. showed increased resistance of chicken respiratory epithelium cultures to infection by a Coronavirus (avian infectious bronchitis virus) after exposure to Vitamin C. However, this animal virus does not share the receptor and pathway with SARS-Cov-2, for which there is no specific evidence regarding potential Vitamin C benefits [117].

In humans, supplementation of vitamin C improves the immune system, preserving the redox integrity of cells as well as protecting from ROS. Vitamin C reduces the risk, the severity, and the duration of different infectious diseases [118,119].

Vitamin C deficiency was historically associated with pneumonia [120,121]. On the other hand, randomized trials demonstrated that supplementation of vitamin C has positive effects on symptoms and on the duration of respiratory tract infections. Additionally, several data suggest that vitamin C can prevent pneumonia and improve its outcome, as well as other infections [122,123,124,125].

Moreover, it has been shown that intravenous vitamin C may reduce inflammation and diminish vascular injury associated with sepsis and ARDS [126,127]. Studies on lung injury induced by sepsis showed that vitamin C reduces the proinflammatory and procoagulant changes that lead to lung damage [126]. Moreover, during infection, vitamin C levels can decrease; therefore, high doses of intravenous Vitamin C administration are required in severe cases, in order to compensate for the high turnover of the vitamin [128].

However, studies on the usefulness of vitamin C for patients with severe pneumonia are limited [129]. A retrospective before–after clinical study showed that a combination of vitamin C, hydrocortisone, and thiamine prevented organ dysfunction and reduced the mortality rate in patients with sepsis [130]. Treatment with the same combination was associated with significantly lower mortality in patients with severe pneumonia and significantly improved their radiologic chest findings [131]. Vitamin C and corticosteroids play in a synergistic way: vitamin C restores glucocorticoid receptor function, and corticosteroids increase the expression of sodium–vitamin C transporter-2 [132,133] Moreover, an experimental study showed that vitamin C and hydrocortisone administered together preserved endothelial integrity [134]. In a recent randomized trial, evaluating patients with sepsis and ARDS, a beneficial effect of high-dose intravenous vitamin C on mortality has been suggested, although no improvement of organ dysfunction scores or change in markers of inflammation and vascular injury were observed [135,136,137]. Moreover, recently, a new clinical trial to investigate the effects of vitamin C infusion for the treatment of severe COVID-19 pneumonia has started [138]. In fact, in the absence of a specific therapy for COVID-19, vitamin C may have effects on this severe viral respiratory tract infection [139]. Moreover, vitamin C increased the resistance to coronavirus and may affect the susceptibility to lower respiratory tract infections under certain conditions [117,139,140,141].

### 5.2. Vitamin D

The biosignaling role of vitamin D in bone metabolism is well known, but over the years, this metabolite has been linked to the risk of developing various pathologies, such as cancer, depression, and infectious diseases. After binding its nuclear receptor, the active metabolite of vitamin D (1,25(OH)2D3 or calcitriol) influences gene transcription, exerting several effects also on the immune and inflammatory response. As recently summarized in a review by Grant et al., Vitamin D would act against respiratory infections through many pathways [142]. The receptor for vitamin D is expressed in respiratory epithelial cells and in macrophages of the respiratory system; furthermore, the 25- hydrolase, which converts vitamin D into its active metabolite, is constitutively expressed in the respiratory epithelium [143]. The in vitro study of Philip et al. showed that in the presence of 1,25(OH)2D3, macrophagic production of catelicidine (like LL-37) is increased. By binding the envelope of influenza A and respiratory syncytial viruses, catelicidins are capable to damage its structure and prevent infection of human cells [144].

Much evidence has also suggested that other biosignaling pathways linked to vitamin D may modulate the inflammatory response depending on both the innate and specific systems. In fact, 1,25(OH)2D3 modulates nuclear factor κB (NF-kB) activity via upregulation of the NF-κB inhibitory protein (IκBα). NF-kB induces the production of many molecules which amplify the inflammatory response (IL-6, IL1-β, TNF-α), stimulate the production, mobilization, and adhesion of inflammatory cells (GM-CSF, IL-4, IL-5, VCAM-1, ICAM-1, E-selectin), and finally influence the production of enzymes such as iNOS, COX-2, PLA2 and determine the production of free radicals causing tissue damage [145,146].

There are many other molecular mechanisms through which vitamin D would be able to stimulate the immune response, reduce the risk of infections, and balance the inflammatory reaction probably in a favorable way for the body. The study of the clinical effects of vitamin D administration in patients with and without demonstrated vitamin D deficiency could be very interesting today in the setting of COVID-19; however, specific data are not yet available.

In the analysis of Monlezun et al. evaluating 14,108 subjects (>16 years of age), after adjusting for confounding factors like season or demographic and clinical data, vitamin D levels <30 ng/mL were associated with 58% higher odds of acute respiratory infection compared to levels ≥30 ng/mL [147].

A meta-analysis of data of 10,933 participants from 25 randomized controlled trials showed that vitamin D administration reduces the risk of acute respiratory tract infections (OR 0.88, 0.81 to 0.96; P for heterogeneity <0.001). In subgroup analysis, protective effects were obtained for participants assuming daily or weekly doses with no supplemental bolus (OR 0.81, 0.72 to 0.91) but not for subjects assuming one or more boli (OR 0.97, 0.86 to 1.10; P for interaction = 0.05). Regarding the daily or weekly administration of vitamin D, benefits were more evident in participants with low calcifediol levels (<25 nmol/L) at baseline (OR 0.30, 0.17 to 0.53) than in subjects with initial levels ≥25 nmol/L (OR 0.75, 0.60 to 0.95; P for interaction = 0.006). Finally, the administration of vitamin D was safe [148].

The hypothesis that vitamin D supplementation can reduce the risk of COVID-19 incidence or mortality should be investigated through large-scale randomized trials. Currently, no data are available on the dosage, method of administration (daily or bolus), and safety in the setting of COVID-19; however, for the moment it is reasonable to focus on the identification and treatment of deficiencies in asymptomatic subjects as well as in patients affected by COVID-19. An Italian study group has proposed a nutritional protocol for patients with COVID-19, which also includes the supplementation of 25-hydroxyvitamin D in the presence of a deficit [149].

## 6. Conclusions

Although orally administered probiotics are not currently an integral part of a specific protocol for the treatment of respiratory viral infections, many studies suggest their potential modulation of the systemic immune system that can improve the response to viruses and balance the inflammatory response. SARS-CoV-2 infects the gastrointestinal tract, causing inflammation of the absorbent mucosa and sometimes diarrhea. Dysbiosis could participate in this scenario, exacerbating the immune response and the production of systemic inflammation mediators. Based on the revised evidence, oral probiotics could therefore play a role in the intestinal and systemic effects of COVID-19. Moreover, inhaled microorganisms could have a more direct action on the respiratory epithelium and on the immune system cells that populate it. In some circumstances, they have been shown to reduce the accumulation of inflammatory cells and facilitate virus clearance. Several nutrients have shown utility in preserving endothelial integrity thanks to the maintenance of oxidative–reductive homeostasis. COVID-19 can induce pulmonary vascular damage and systemic hypercoagulability. During the pandemic, as in all other circumstances, it is reasonable to recommend a proper nutrition rich in antioxidant nutrients. Vitamin C and D play a well-proven role in the immune system. However, it is not known whether a supplemental dose of these vitamins administered to patients without their deficiency would result in a benefit. Specific clinical studies are underway on the intra-venous administration of vitamin C in hospitalized COVID-19 patients. Vitamin D deficiency has been associated with increased susceptibility to respiratory infections, therefore it is reasonable, even in the absence of specific data, to administer vitamin D to healthy individuals and COVID-19 patients.

While diet, nutritional supplements, and similar interventions show great promise for preventing and managing COVID-19, it is also true that strong clinical research data are required to support any such claim. Otherwise, we risk the emergence of gurus or other more or less well-meaning experts aiming at speculating on the appeal of these interventions for laypersons [150].

## Figures and Tables

**Table 1:** Registered trials evaluating the possible benefits of probiotics administration in COVID-19 patients.

Study Title	Study Type and Design	Study Design	Outcomes	Reference
Evaluation of the Probiotic *Lactobacillus coryniformis* K8 on COVID-19 Prevention in Healthcare Workers NCT04366180	Interventional, Randomized Active, recruiting	“To evaluate the effects of *Lactobacillus coryniformis* K8 consumption on the incidence and severity of Covid-19 in health workers exposed to the virus. This is a preventive study. Estimated enrolment: 314 participants”	Incidence of SARS CoV-2 infection in healthcare workers Incidence of hospital admissions caused by SARS-CoV-2 infection Incidence of intensive care unit (ICU) admissions caused by SARS-CoV-2 infection Incidence of oxygen support requirement caused by SARS-CoV-2 infection Incidence of gastrointestinal symptoms caused by SARS-CoV-2 infection Days with body temperature >37.5 °C Days with cough Days with fatigue Medical treatment	[91]
Bacteriotherapy in the Treatment of COVID-19 (BACT-ovid) NCT04368351	Observational, Retrospective Active, not recruiting	“Observational, retrospective, non-profit study on the adjuvant use of bacteriotherapy in the early control of disease progression in patients affected by COVID-19 and treated with the current standard of care on the basis of the ad interim Italian guidelines. Estimated enrolment: 70 participants”	Delta of time of disappearance of acute diarrhea Delta in the number of patients requiring orotracheal intubation despite treatment Delta of crude mortality Delta of length of stay for patients in hospital	[92]
Oxygen–Ozone as Adjuvant Treatment in Early Control of COVID-19 Progression and Modulation of the Gut Microbial Flora (PROBIOZOVID) NCT04366089	Interventional, Randomized Active, recruiting	“Interventional, non-pharmacological, open, randomized, prospective, non-profit study on the adjuvant use of oxygen–ozone therapy plus probiotic supplementation in the early control of disease progression in patients with COVID-19. Contextually, all patients are treated with the current standard of care on the basis of the interim Italian guidelines. The main purpose of the study is to evaluate the effectiveness of an ozone therapy-based intervention (accompanied by supplementation with probiotics) in containing the progression of COVID-19 and in preventing the need for hospitalization in intensive care units.”	Delta in the number of patients requiring orotracheal intubation despite treatment Delta of crude mortality Delta of length of stay for patients in hospital Delta in the value of interleukin (IL)-1 Delta in the value of IL-6 Delta in the value of IL-10 Delta in the value of tumor necrosis factor (TNF)-alpha Delta in the value of cluster of differentiation (CD)4+ CD38/human leukocyte antigen-DR isotype (HLA-DR) Delta in the value of CD8+ CD38/HLA-DR Delta in the value of faecal calprotectin Delta in the value of lipopolysaccharide (LPS) Delta in the value of zonulin Delta in the value of alpha1-antitrypsin	[93]

**Table 2:** Main nutraceuticals and supplements with potential role in countering COVID-19 pathways.

Main Nutraceuticals and Supplements	Pathway Hypothesized Against COVID-19	Supporting Literature
Vitamin E, Vitamin C, Carotenoids, Minerals (Zn, Mn, Cu, Se) Polyphenols	Inflammatory cascade and hypercoagulation by anti-inflammatory and antioxidant activities (in COVID-19 pathways, the endothelium target could be relevant)	[85,86,98]
Polyphenols (flavonoids, phenolic acids, stilbenes, lignans)	Platelet aggregation and pro-thrombotic activity by suppression of thrombin and factor Xa; endogenous platelet-derived NO and superoxide production; endothelial synthesis of NO, NO signaling pathways in endothelial cells improving endothelial function and NO-dependent relaxation; modulates production of cytokines and expression of pro-inflammatory genes Antiviral effect for several viruses (not proved for SARS-CoV-2).	[95,96,97,99,100]
Curcumin	Binds to the target receptors of SARS-CoV-2	[106]
Combination of vitamin C, curcumin, and glycyrrhizic acid	Interferons production with effects on inflammatory response.	[107]
Lianhuaqingwen (Chinese patent medicine composed of 13 herbs)	SARS-CoV-2 replication; pro-inflammatory cytokines (TNF-α, IL-6, CCL-2/MCP-1, and CXCL-10/IP-10).	[108]
Vitamin C	Fundamental for the structural organization of the epithelial and endothelial barriers; fundamental for phagocytosis and chemotaxis; protection from ROS injury; intravenous administration against inflammation and vascular injury in sepsis and ARDS; susceptibility and outcome of low respiratory tract infections.	[98,99,105,106,107,108,109,110,122,124]
Vitamin D	Macrophagic production of catelicidine; regulation of NF-kB activity levels of IL-6, IL1-β, TNF-α and production of GM-CSF, IL-4, IL-5, VCAM-1, ICAM-1, E-selectin; daily or weekly dose showed protective effects against acute respiratory infections.	[127,128,129,130,131]
